# Technical Aspects and Development of Transcatheter Aortic Valve Implantation

**DOI:** 10.3390/jcdd9080282

**Published:** 2022-08-22

**Authors:** Klemen Steblovnik, Matjaz Bunc

**Affiliations:** Department of Cardiology, University Medical Centre Ljubljana, 1000 Ljubljana, Slovenia

**Keywords:** aortic stenosis, TAVI, materials, technology

## Abstract

Aortic stenosis is the most common valve disease requiring surgery or percutaneous treatment. Since the first-in-man implantation in 2002 we have witnessed incredible progress in transcatheter aortic valve implantation (TAVI). In this article, we review the technical aspects of TAVI development with a look at the future. Durability, low thrombogenicity, good hydrodynamics, biocompatibility, low catheter profile, and deployment stability are the attributes of an ideal TAVI device. Two main design types exist—balloon-expandable and self-expanding prostheses. Balloon-expandable prostheses use a cobalt-chromium alloy frame providing high radial strength and radiopacity, while the self-expanding prostheses use a nickel-titanium (Nitinol) alloy frame, which expands to its original shape once unsheathed and heated to the body temperature. The valve is sewn onto the frame and consists of the porcine or bovine pericardium, which is specially treated to prevent calcinations and prolong durability. The lower part of the frame can be covered by polyethylene terephthalate fabric or a pericardial skirt, providing better sealing between the frame and aortic annulus. The main future challenges lie in achieving lower rates of paravalvular leaks and new pacemaker implantations following the procedure, lower delivery system profiles, more precise positioning, longer durability, and a good hemodynamic profile. Patient-specific design and the use of autologous tissue might solve these issues.

## 1. Introduction

The stenosis of the aortic valve is a common, mostly degenerative process that is becoming increasingly prevalent with an ageing population [1]. No medical therapy is available to improve the prognosis of patients with aortic stenosis [2,3] and, until two decades ago, surgery was the only treatment option. Surgical aortic valve replacement requires the use of a heart–lung bypass machine and carries the risk of major complications and a prolonged recovery. Therefore, aortic stenosis treatment was not available to patients with a prohibitively high surgical risk, such as old and fragile patients or those with significant comorbidities. To overcome this issue a new, less invasive method—transcatheter aortic valve implantation (TAVI) was developed. The beginnings of this procedure date to the late 1980s when dilatation of the degenerated aortic valve with a balloon—a balloon valvuloplasty—was first performed [4]. However, the lack of survival benefit and early restenosis were important limitations [5,6]. The concept of transcatheter implantation of a large-size metallic stent with a mounted prosthesis became promising. In 1989, Henning-Rud Andersen implanted a balloon-expandable prosthesis consisting of a hand-made mesh with a porcine valve into pigs [7]. The first-in-men use of this concept was performed by Philip Bonhoeffer, who implanted a bovine jugular vein conduit attached to a platinum-iridium stent in a right ventricle to pulmonary artery conduit in 2000 [8]. Additionally, in the year 2000, Alain Cribier performed the first successful transcatheter implantation of a balloon-expandable prosthesis in the aortic position in a sheep, consisting of a stainless-steel stent with a polyurethane valve [9]. The same type of bioprosthesis was used in the first-in-men TAVI, which was also successfully performed by Cribier in 2002 [10]. In 2004, the first commercial devices became available and since then TAVI has revolutionized the treatment of patients with aortic stenosis. From the early stages, significant progress has been made in terms of catheter size, deliverability, prosthesis durability, and paravalvular leakage prevention. In this article, we review the technical aspects of TAVI development with a look at the future.

## 2. Method Description and Requirements

The minimal invasiveness of TAVI, which brings the biggest benefit for the patients, is achieved by the delivery and deployment of the bioprosthesis over a catheter system. The majority of procedures are performed using a femoral artery as an access point. Transfemoral access is superior to alternative access options [11]. However, other percutaneous (axillary, subclavian, or carotid artery) or surgical (heart apex or ascending aorta) accesses are also available. There are also reports of transcaval and subxiphoid TAVI implantations but these procedures are not broadly accepted [12]. The surgical access is less beneficial than the transfemoral and is related to higher postprocedural mortality [13].

For a transfemoral TAVI, the femoral artery is punctured and a wire is inserted retrogradely into the aorta. Using the Seldinger technique [14] a 14–20 French sheath, which facilitates the passage of the delivery catheter, is positioned with the tip in the distal aorta. After removing the tapered tip dilator from the sheath, a wire is inserted retrogradely through the aortic valve into the left ventricle. The wire acts as a railway for the delivery catheter with the bioprosthesis at its distal end. Under fluoroscopic guidance the prosthesis mounted onto a metallic frame is deployed at the aortic position compressing the native valve so that sealing is achieved without suturing.

The development of a TAVI device with a catheter delivery system and the bioprosthesis crimped at the distal end is technically demanding. A set of guidelines for heart valves implanted with transcatheter techniques was developed by the International Organization for Standardization (ISO 5840-3).

Many designs for TAVI bioprosthesis have been developed and tested. Generally, the device consists of a metallic frame with a valve mounted in the center. Because of a transcatheter delivery system the device must be crimped outside of the patient and mounted onto a catheter. Regarding deployment two main design types of the bioprosthesis exist—balloon-expandable and self-expanding. The two most common platforms are Sapien 3/Ultra (Edwards Lifesciences Corporation, Irvine, CA, USA) and Evolut^TM^ R/PRO/PRO+ (Medtronic, Minneapolis, MN, USA). Sapien 3/Ultra is a balloon-expandable and Evolut^TM^ R/PRO/PRO+ is a self-expanding device. Regarding the position of valve leaflets in relation to the failed native valve or bioprosthesis annulus, TAVI device design can be intra-annular or supra-annular. Aortic annulus is the narrowest part of the blood flow and using a prosthesis with intra-annular design can lead to small effective orifice area. In supra-annular prosthesis design, however, the leaflets are sewn to the frame above the annulus and are unconstrained by the native/failed bioprosthetic annulus, which might lead to a higher effective orifice area [15,16,17,18]. However, there is conflicting real-world data, showing that both supra- and intra-annular self-expanding devices demonstrate superior hemodynamics compared to balloon-expandable devices in patients with small aortic annulus [19].

## 3. Frame

### 3.1. Balloon Expandable Devices

Balloon-expandable devices are crimped outside of the patient and mounted onto a catheter. Inside the aorta, before reaching the aortic valve, the device is mounted onto a semi-compliant balloon. Once the device is positioned under fluoroscopic guidance inside the failed native aortic valve or bioprosthesis, the fast ventricular pacing is started. Pacing can be achieved using a temporary pacemaker lead introduced to the right ventricle or over the stiff guiding wire in the left ventricle. At pacing frequencies above 175 beats per minute, cardiac output starts to diminish significantly and a controlled temporary cardiac arrest is achieved. During fast ventricular pacing, the balloon is inflated and the metallic frame of the device is expanded. Short inflation (usually 3–5 s) is followed by balloon deflation and the discontinuation of the fast ventricular pacing. At this point, spontaneous circulation is restored and the balloon is removed over the guiding wire while the TAVI device stays in place. The high radial force is needed for the device to maintain its structure and good contact with the aortic annulus, preventing paravalvular regurgitation. The frame of Sapien 3/Ultra, which is the most commonly used balloon-expandable device, is made of cobalt-chromium (Co–Cr) alloy. Co–Cr alloys have been used in the production of medical and dental implants due to their high corrosion-resistance, biocompatibility, and good mechanical properties similar to stainless steel [20,21,22]. Compared to stainless steel Co–Cr alloys have a higher elastic modulus, yield strength, tensile strength, and density, which offers better radial strength, radio-opacity, and magnetic resonance imaging compatibility [20,23,24]. The hardness of Co–Cr alloys ranges from 550 to 800 MPa, and tensile strength ranges from 145 to 270 MPa [22]. This allows frame struts to be thinner, which is important to achieve a smaller delivery system profile.

A numerical analysis of the radial force has revealed that the radial force may vary, and it is higher at the smaller annular sizes. The radial force of self-expanding devices depends mainly on the left ventricular outflow tract (and annulus) diameter, while the radial force in the balloon-expandable devices is influenced by both the geometry and stiffness of the host tissue [25].

### 3.2. Self-Expanding Devices

Self-expanding devices are crimped onto the distal tip of a delivery catheter just before implantation and are held in the compressed shape by a capsule. During the deployment, which does not require fast ventricular pacing accompanied by a transient cardiac arrest, the capsule is slowly removed under the fluoroscopic guidance and the frame of the device expands. This kind of deployment allows for the resheathing and repositioning of the device if the position is not optimal. Material that enables the self-expanding nature of these devices is Nitinol—an alloy of nickel and titanium. A transformation between a higher temperature austenite phase and a lower temperature martensite phase makes this alloy unique due to its superelastic properties and shape memory [26]. Austenite is stable at higher temperatures and can be reversely transformed to martensite, which is stable at lower temperatures, by an external force, temperature change, or both. The transformation from austenite to martensite due to external stress above the transformation temperature is called superelasticity [27]. This transformation is reversed if the external stress is removed. Nitinol transformation that occurs due to temperature change is called shape memory. The temperature at which the shape transformation takes place is closely related to the amount of nickel and titanium in the alloy as a 1% shift in the amount of nickel or titanium results in a 100 °C change in the alloy-transformation temperature [28]. The usual Nitinol alloy consists of 50.8% nickel and 49.2% titanium. After hot and cold working shape setting takes place in specific temperature and strain conditions, and the “remembered” shape is formed. Superelasticity, shape memory, and biocompatibility make Nitinol a favorable material in medical devices, such as self-expanding peripheral stents, TAVI devices, orthopedic and dental prosthetics. With the transition point at room temperature, TAVI devices with Nitinol frames can be compressed and stabilized by a capsule at the distal end of the catheter. Removing the capsule at the body temperature causes the Nitinol frame to acquire its remembered shape and maintain its radial force. However, nickel is known to trigger allergic [29], toxic [30], and possibly carcinogenic effects [31]. Thus, the release of nickel has been a concern in medical devices. With surface passivation and electrochemical polishing, a stable titanium oxide (TiO_2_) layer is formed on the surface which prevents nickel release from the Nitinol devices [32]. In vitro and in vivo studies showed that the biocompatibility of Nitinol was good even in patients with nickel hypersensitivity [33]. Nevertheless, hypersensitivity to nickel or titanium is still a contraindication for Nitinol frame TAVI device implantation. The implantation of a TAVI prosthesis triggers a systemic inflammatory response syndrome (SIRS) in around one-third of patients [34]. The occurrence of SIRS is associated with increased mortality [35]. A neutrophil-to-lymphocyte ratio can be used as a prognostic marker in TAVI patients [36]. TAVI-associated SIRS is more extensive with self-expanding compared to balloon-expandable devices [37]. This could be partially explained by more pronounced endothelial injury (due to oversizing and continuous pressure to surrounding tissues) and an increased inflammatory reaction to a foreign body placement (due to the presence of residual cells, DNA, and the alpha-Gal epitope in porcine-derived materials) of self-expanding devices [37]. Endothelial injury and foreign material placement trigger the immune response, which begins with fibrinogen adsorption and platelet activation followed by recruitment of circulating leukocytes [37]. Titanium content enhances fibrinogen adsorption [38], which could partially explain a pronounced immune response following TAVI with self-expanding devices. Chronic inflammation might also be one of the mechanisms for valve degeneration and malfunction.

The size of self-expanding devices is ideally slightly bigger than the actual aortic annulus—optimal oversizing produces a consistent radial force and better sealing. However, constant radial force creates pressure on the neighboring structures, such as the heart conduction system, especially the atrioventricular node and bundle of His. The damage to the heart conduction system can cause bradycardic heart rhythm disturbances, resulting in the need for a permanent cardiac pacemaker. Indeed, the rate of new, postprocedural pacemaker implantation is higher in self-expanding devices than in balloon-expandable devices (17.4 vs. 6.5% for CoreValve^TM^/Evolut^TM^ R/PRO vs. Sapien 3, respectively) [39,40,41]. This association can be explained by the anatomic relationship between the heart conduction system and the frame of TAVI bioprosthesis. The compact part of the atrioventricular node lies in the right atrial wall region called the triangle of Koch in close proximity to the atrioventricular part of the membranous septum, which on the ventricular side represents a part of the left ventricular outflow tract between the right and non-coronary cusp of the aortic valve. The atrioventricular branch (bundle of His) perforates the membranous septum and splits into the right and left bundle branches. The left bundle branch usually lies just below the endocardium of the interventricular septum representing a part of the left ventricular outflow tract. However, the exact anatomy of the atrioventricular branch varies [42]. Calcifications in the left ventricular outflow tract, especially below the non-coronary and right coronary cusp are associated with a postprocedural atrioventricular block [43].

### 3.3. Frame Shape and Size

TAVI platforms use different frame shapes and are produced in several sizes to accommodate the anatomy of the patients. Appropriate frame size is determined mainly by aortic annulus diameter, perimeter, and area. However, not only size but also careful shape consideration is necessary before every implantation. Aortic root diameter, sino-tubular junction and ascending aorta diameters, aortic angulation, and coronary ostia height have to be considered in device selection. To reliably determine these anatomical parameters an ECG-synchronized CT angiography of the aortic root and heart should be routinely performed [44]. Usually, semi-automatic software is used for measurements. However, in the last few years, fully automatic software (HeartNavigator3, Philips, Amsterdam, The Netherlands) is emerging. It allows for fast, user-friendly, and reliable measurements [45]. Artificial intelligence-based automatic segmentation software (Heart AI, Laralab, München, Germany) combined with 3D printing can also be used to simulate TAVI procedures. In challenging cases, such as valve-in-valve TAVI procedures, preprocedural planning using 3D printed models may be very helpful [46].

Valve-in-valve procedures are used to treat failed surgical or percutaneous bioprostheses. However, in such procedures, the degenerated prosthesis is not extracted from the patient and the frame of the new prosthesis has to be smaller than that of the failed one. Therefore the effective orifice area of the inserted prosthetic valve is too small in relation to the body size, which leads to higher-than-expected gradients through a normal functioning prosthesis—patient–prosthesis mismatch [47]. This phenomenon is gaining in clinical importance due to the increase in longevity and the use of bioprostheses at a younger patient age. To address this issue, new methods of bioprosthesis retrieval are being developed. A system called exchangeable-TAVI (e-TAVI) is using an electromagnetic catheter to remove and retrieve a failed exchangeable prosthesis, followed by the immediate deployment of a new prosthesis. According to simulations a combination of magnetic and mechanical coupling would be needed [48]. This concept, however, is not yet implemented in clinical practice. Another technique, bioprosthetic valve fracture (BVF) performed as a part of the valve-in-valve TAVI has been increasingly used to avoid patient–prosthesis mismatch in certain types of small surgical bioprostheses [49]. With BVF, the operator “cracks” the ring of the surgical bioprosthesis by using a high-pressure noncompliant transcatheter balloon, either before or after implanting the transcatheter valve. This allows the implantation of larger TAVI prostheses with better hemodynamic performance [50,51]. In case of valve-in-valve treatment, a variable level of the two bioprostheses overlapping is obtained, which creates an oval “neoskirt”. The long-term hemodynamic effect of such a “neoskirt” is not known.

Coronary ostia height; the sinus of the Valsalva diameter; in cases of valve-in-valve procedures, also valve-to-coronary (VTC) distance; and the type of failed bioprosthesis are important parameters, which could predict periprocedural coronary ostia obstruction [52]. Several techniques can be used to prevent this, often lethal, complication. The use of devices with low frame height, slightly lower implantation depth, the placement of a safety wire, and an undeployed stent in a coronary artery, as well as the deployment of a coronary stent extending from the proximal portion of a coronary artery cranially and parallel to the TAVI bioprosthesis (“chimney stenting technique”) [53] and bioprosthetic or native aortic scallop intentional laceration to prevent iatrogenic coronary artery obstruction (BASILICA) [54] can be used as preventive measures. Coronary artery intubation can be challenging after TAVI. Patients who might need coronary artery access in the future (due to coronary artery disease or young age) can benefit from lower frame height and large cell design, which allow for easier coronary ostia access [55]. Not only frame height and frame cell design but also commissural alignment and valve-in-valve procedure greatly impact coronary access after TAVI [56].

### 3.4. Sealing

Since a TAVI bioprosthesis is not sewn to the aortic annulus, adequate sealing has to be achieved by the compression of the frame against the native structures (annulus and leaflets). However, at least mild paravalvular regurgitation often exists [57], especially if there is a large aortic valve calcification volume [58]. Moderate or worse paravalvular regurgitation is associated with an increased risk of all-cause and cardiovascular death, rehospitalization, and reintervention at two years [59]. With improvements in preprocedural planning (the routine use of 3D computed tomography for valve sizing and selection) and improvements in technology (repositionability, sealing “skirts”) the incidence of postprocedural paravalvular regurgitation has decreased [60]. New generation TAVI devices (such as Edwards Lifesciences (Irvine, CA, USA) Sapien 3 Ultra, Abbot Laboratories (Chicago, IL, USA) Navitor^TM^, Medtronic (Minneapolis, MN, USA) Evolut^TM^ PRO+, and Boston Scientific (Marlborough, MA, USA) ACURATE neo2^TM^) use a polyethylene terephthalate or porcine pericardium “skirt” covering the inflow part of the frame to diminish paravalvular regurgitation.

## 4. Valve

Native aortic valve tissue architecture with its three layers—fibrosa, spongiosa, and ventricularis—provides for high compliance along its radial direction, which allows leaflets to be stretched in diastole while circumferential stiffness is preserved for supporting the high transvalvular pressure [61]. Current TAVI devices are so-called bioprostheses, which means that the valve leaflets are made from biological material and are handsewn to the metallic frame. Bovine or porcine pericardium xenografts are used in FDA and CE-approved TAVI devices. Historically bovine pericardium has been identified as a biomaterial of choice due to its superior mechanical properties. However, with the development of TAVI techniques striving for minimal invasiveness the size of the delivery catheter became increasingly important. The minimal diameter of a crimped TAVI device is largely determined by the leaflet thickness. Porcine pericardium (0.14–0.20 mm) is thinner than bovine pericardium (0.32–0.42 mm) but also stiffer and less extensible with similar tensile strength [62]. Degenerative and inflammatory processes leading to native valve failure also take place with bioprostheses but at a faster pace, especially in younger patients [63,64]. Fibrocalcification, thrombosis, and immune rejection have been identified as the main degeneration pathophysiological processes [65]. Pericardium used in TAVI device manufacturing is fixed with glutaraldehyde to mask antigen and avoid immune rejection. However, glutaraldehyde exacerbates the passive calcification process [66] and may not completely remove the antigenicity of the bioprosthetic tissue. To reduce calcification and leaflet degeneration, the pericardium is processed with functional group capping (aldehyde reduction), glycerolization, ethylene oxide sterilization [67,68]. Crimping—a TAVI-specific feature which causes significant mechanical stress to the leaflets and may further promote the degenerative processes [69]. The hydrogel hybridization of glutaraldehyde-crosslinked porcine pericardium is being tested to produce a pre-mounted bioprosthetic heart valve to avoid the need for crimping [70]. Dry tissue technology to allow for a pre-mounted, pre-crimped, and pre-loaded prosthesis that is sterilized and ready for use (Colibri, Colibri Heart Valve LLC, Broomfield, CO, USA) is another innovative solution that needs clinical approval [71]. All biological prostheses tend to deteriorate with time, which eventually requires a reintervention. This pathophysiological process, called the bioprosthetic valve dysfunction, is not uniform and includes structural valve deterioration (SVD), non-structural valve deterioration, thrombosis, and endocarditis [72]. Although long-term data on SVD in TAVI patients is scarce, there is evidence that there is a similar rate of SVD in surgical and TAVI patients at five years [73]. Registry data show low rates of SVD even after five years [74,75]. The NOTION trial—the first randomized trial on bioprosthetic valve durability in patients with low surgical risk of mortality—even showed a lower rate of SVD in TAVI compared to surgical patients at 8 years (13.9% vs. 28.3%; *p* = 0.0017) and the risk of bioprosthetic valve failure was similar (8.7% vs. 10.5%; *p* = 0.61) [76]. Even though long-term durability data, critically important before providing TAVI to younger patients, is lacking, we can presume that TAVI prostheses will be subjected to similar material-specific constraints as surgical bioprostheses. To overcome this issue, tissue engineering has been used to produce a valve with self-repair and remodeling capacities. Decellularized homo- and xenografts, in vitro-grown tissue-engineered matrices, bioresorbable polymers, pre-seeded biodegradable polymer-based vascular grafts using autologous bone marrow cells, personalized cardiovascular tissues 3D (bio)printing, and other methods have been studied [77,78,79]. However, none of these concepts is present in current everyday practice.

## 5. Delivery System and Access

Although the transfemoral approach is considered the golden standard for TAVI, vascular complications are still the most common complications of the procedure with an incidence of major complications between 1% and 10% [80,81,82]. Female gender, obesity, peripheral artery disease, femoral artery diameter, sheath size, calcification, and use of dual antiplatelet therapy have been associated with a higher incidence of vascular complications [83,84,85,86,87,88]. Careful preprocedural planning containing the routine use of CT angiography determining best puncture site, minimal lumen diameter, severity and length of stenoses, degree of calcifications, and tortuosity is necessary to avoid vascular complications [44]. Semi-automatic software (such as 3mensio Vascular, Pie Medical Imaging, Maastricht, The Netherlands) can be used for reconstruction and measurements.

Technological advances have enabled smaller delivery catheter profiles, which enables transcatheter access to be used in more than 95% of patients. Minimal vessel diameter for low delivery profile systems with an integrated sheath (such as Medtronic Evolut^TM^ PRO+ or Abbot Navitor^TM^) is 5.0 mm. Delivery systems with an integrated sheath contain a sheath with an equal diameter to that of the bioprosthesis capsule at the tip of the catheter. This avoids the need for a special introducer sheath. With other devices, introducer sheaths are used to enable access to a delivery system through iliac and femoral arteries, where the vessel diameter is usually the smallest. Sheath shafts are coated with a hydrophilic polymer to diminish friction damage caused by interaction between the vessel wall and the catheter. Although greatly increasing deliverability, cases of hydrophilic polymer coating embolization have been described [89]. Multiple layers of dopamine-modified hyaluronic acid and chitosan show promising results for the further reduction of friction and vessel wall damage [90]. Expandable sheaths (such as Edwards eSheath or Boston Scientific iSleeve^TM^) have a folded wall that expands with the passage of the delivery catheter. In this way, the unexpanded sheath has a low profile (14–16 French) and can easily cross the narrow parts of the femoral or iliac arteries. As the delivery catheter advances, the sheath expands in diameter, thus avoiding friction-associated damage to the arterial wall.

Thus, the calcific lesions of femoral or iliac arteries might be uncrossable even with low-profile catheters, especially if circular calcifications are present. These transform the vessel into a “stiff tube”, which limits arterial expansion to accommodate introducer sheaths or TAVI delivery systems, increasing the risk of dissection or perforation [91]. Recently, intravascular lithotripsy (Schockwave^TM^ catheter, Schockwave Medical Inc., Santa Clara, CA, USA) has emerged as a treatment option for heavily calcified stenotic lesions, facilitating the transfemoral TAVI approach [92]. The intravascular lithotripsy system uses an over-the-wire balloon catheter with emitters enclosed inside of the balloon. At the desired location the balloon is inflated to 4–6 atm so that good apposition with the arterial wall is achieved. The generator connected to the catheter then produces electrical impulses that discharge at the emitters, causing fluid vaporization and rapidly expanding bubbles. This creates a localized field effect around the lithotripsy balloon ranging into the arterial media. Sonic waves damage uncompliant structures, such as calcifications, but do not injure soft vascular tissues. In this way, intravascular lithotripsy converts a non-compliant calcified part of the arteries into more compliant structures with cracked calcium fragments staying inside the vessel wall.

In severe calcified aortic stenosis and specific anatomies, such as a horizontally positioned ascending aorta, the passage of the delivery catheter through the aortic valve might be challenging. To improve the crossing of the native aortic valve and enable coaxiality (less contact between the delivery catheter and aortic wall) some delivery catheters (such as Edwards Commander Delivery System) are steerable.

## 6. Future of TAVI

Aiming to achieve native valve-like hydrodynamics, long durability, retrievability, biocompatibility, low thrombogenicity, minimal paravalvular leakage, low new pacemaker implantation rate, and lower delivery system profiles a TAVI device of the future will possibly be custom-made, respecting individual patient anatomy. Delivered by a minimally invasive method, it might use tissue-engineered, possibly in vivo 3D printed scaffolds that will be populated by autologous cells. Often coexisting with mitral or tricuspid valve disease, TAVI may be complemented with other rapidly developing transcatheter treatment methods [93,94]. With growing knowledge patient specific medical therapy could be tailored diminishing the risk of valve thrombosis, degeneration, or heart failure. Although already potentially being a cost-effective treatment option [95], with exponential growth TAVI should become much less expensive and available to the patients all over the world.

## Data Availability

Not applicable.
